# Efficacy of brief behavioral counselling by allied health professionals to promote physical activity in people with peripheral arterial disease (BIPP): study protocol for a multi-center randomized controlled trial

**DOI:** 10.1186/s12889-016-3801-7

**Published:** 2016-11-09

**Authors:** Nicola W. Burton, Zanfina Ademi, Stuart Best, Maria A. Fiatarone Singh, Jason S. Jenkins, Kenny D. Lawson, Anthony S. Leicht, Yorgi Mavros, Yian Noble, Paul Norman, Richard Norman, Belinda J. Parmenter, Jenna Pinchbeck, Christopher M. Reid, Sophie E. Rowbotham, Lisan Yip, Jonathan Golledge

**Affiliations:** 1 The University of Queensland School of Human Movement & Nutrition Sciences, St Lucia, Brisbane, QLD 4072 Australia; 2 University of Basel Institute of Pharmaceutical Medicine, Basel, Switzerland; 3 University of Monash Department of Epidemiology and Preventive Medicine, Melbourne, 3004 VIC Australia; 4 Queensland Research Centre for Peripheral Vascular Disease; College of Medicine and Dentistry, James Cook University, Townsville, QLD 4811 Australia; 5 Exercise, Health and Performance Faculty Research Group, Faculty of Health Sciences, University of Sydney, Sydney, NSW 2141 Australia; 6 Vascular Surgery The Royal Brisbane and Women’s Hospital, Herston, QLD 4059 Australia; 7 Centre for Health Research, School of Medicine, Western Sydney University, Sydney, NSW 2753 Australia; 8 Centre for Research Excellence in Chronic Disease Prevention, Australian Institute for Public Health and Tropical Health and Medicine, James Cook University, Townsville, QLD 4811 Australia; 9 Sport and Exercise Science, College of Healthcare Sciences, James Cook University, Townsville, QLD 4811 Australia; 10 Exercise, Health and Performance Research Group, Faculty of Health Sciences, University of Sydney, Sydney, NSW 2141 Australia; 11 Surgery Fremantle Hospital, The University of Western Australia, Crawley, WA 6009 Australia; 12 School of Public Health, Curtin University, Perth, WA 6845 Australia; 13 Department of Exercise Physiology, Faculty of Medicine, University of New South Wales, Sydney, NSW 2052 Australia; 14 School of Public Health and Preventive Medicine, Monash University, Melbourne, VIC 3004 Australia; 15 The University of Queensland School of Medicine, Herston, QLD 4006 Australia; 16 The Royal Brisbane and Women’s Hospital, Herston, QLD 4029 Australia; 17 Department of Vascular and Endovascular Surgery, The Townsville Hospital, Townsville, QLD 4811 Australia

**Keywords:** Exercise, Physical inactivity, Intermittent claudication, Cardiovascular disease, Health counselling, Quality of life, Cost effectiveness, Biomarkers, Multi-disciplinary, Behavioral health

## Abstract

**Background:**

Physical activity is recommended for people with peripheral arterial disease (PAD), and can improve walking capacity and quality of life; and reduce pain, requirement for surgery and cardiovascular events. This trial will assess the efficacy of a brief behavioral counselling intervention delivered by allied health professionals to improve physical activity in people with PAD.

**Methods:**

This is a multi-center randomised controlled trial in four cities across Australia. Participants (*N* = 200) will be recruited from specialist vascular clinics, general practitioners and research databases and randomised to either the control or intervention group. Both groups will receive usual medical care, a written PAD management information sheet including advice to walk, and four individualised contacts from a protocol-trained allied health professional over 3 months (weeks 1, 2, 6, 12). The control group will receive four 15-min telephone calls with general discussion about PAD symptoms and health and wellbeing. The intervention group will receive behavioral counselling via two 1-h face-to-face sessions and two 15-min telephone calls. The counselling is based on the 5A framework and will promote interval walking for 3 × 40 min/week. Assessments will be conducted at baseline, and 4, 12 and 24 months by staff blinded to participant allocation. Objectively assessed outcomes include physical activity (primary), sedentary behavior, lower limb body function, walking capacity, cardiorespiratory fitness, event-based claudication index, vascular interventions, clinical events, cardiovascular function, circulating markers, and anthropometric measures. Self-reported outcomes include physical activity and sedentary behavior, walking ability, pain severity, and health-related quality of life. Data will be analysed using an intention-to-treat approach. An economic evaluation will assess whether embedding the intervention into routine care would likely be value for money. A cost-effectiveness analysis will estimate change in cost per change in activity indicators due to the intervention, and a cost-utility analysis will assess change in cost per quality-adjusted life year. A full uncertainty analysis will be undertaken, including a value of information analysis, to evaluate the economic case for further research.

**Discussion:**

This trial will evaluate the efficacy and cost-effectiveness of a brief behavioral counselling intervention for a common cardiovascular disease with significant burden.

**Trial registration:**

ACTRN 12614000592640 Australian New Zealand Clinical Trials Registry. Registration Date 4 June 2014.

**Electronic supplementary material:**

The online version of this article (doi:10.1186/s12889-016-3801-7) contains supplementary material, which is available to authorized users.

## Background

Peripheral artery disease (PAD) is the narrowing or blockage of the lower limb arteries usually due to atherosclerosis and associated thrombosis [1]. This reduces blood flow to the limbs, with the most recognised symptom being leg pain that typically presents during walking, worsens with exertion, and is relieved by rest (intermittent claudication: IC). Maximal walking capacity in people with PAD is less than 50 % of that in age-matched controls, and the functional limitations are similar to those seen in people with severe heart failure [2]. People with PAD and IC typically avoid walking and related activities, which constrains social and role functioning and activities of daily living, and precipitates poor quality of life and accelerated functional decline [3]. This, in turn, predisposes them to high health service utilisation and associated costs [4–11], and premature mortality [12]. As an indicator of systemic atherosclerosis, PAD is positively associated with other cardiovascular diseases, coronary events and stroke [13], with one meta-analysis suggesting a two-fold increased risk of mortality, cardiovascular mortality and major coronary events over 10 years [14]. Coronary heart and cerebrovascular events account for ~65 % of deaths in people with PAD, with a two year prospective study suggesting a 70 % increased risk of cardiovascular events and an 80 % increased risk of death compared to people with coronary artery disease alone [13].

PAD is a common chronic disease associated with ageing. Prevalence has been reported as 8.3 % in people aged 60–69 years [6], 14.5 % in those aged >70 years [15] and 40 % of people aged >80 years [16]. The economic burden of PAD is substantial, as reflected by expensive vascular surgical interventions aimed at opening or bypassing blocked arteries, and recurring hospitalisations [7]. A total of USD$4.37 billion was spent on PAD-related treatment in 2001, with 88 % for inpatient care [17]. Prior lower limb revascularisation is associated with high hospitalisation costs and rates of subsequent procedures during the following 2 years [7]. Rates of surgical intervention are increasing dramatically: There was a 43 % increase between 2000 and 2008 in Australia which far exceeds the estimated growth in the number of people with PAD [18]. Such interventions have limited durability, are expensive to perform and their value in providing sustained improvement in quality of life and reducing subsequent morbidity and mortality is not well established [19, 20]. Given the high prevalence and burden of PAD, and the large expense and limited durability of surgical interventions, there is an urgent need to identify effective, affordable and sustainable alternative management options.

International guidelines for PAD management recommend exercise [21–24], and typically identify three 40-min sessions per week of interval walking at maximum tolerable speed (i.e., moderate pain level with rest periods) [2, 25]. There is good evidence that supervised exercise is as effective as surgical procedures in improving walking ability in patients that have IC [20, 26, 27]. Supervised exercise programs have been shown to improve walking capacity, functional mobility, maximal walking distance, pain symptoms and health-related quality of life; and to reduce leg pain and cardiovascular risk [20, 28–32]. People with PAD who are physically active have a lower average annual functional decline than those who are inactive [33], and a significantly lower five-year mortality risk [12, 34]. Reduced daily sitting time is associated with a reduced decline in the six minute walk test [35], with improvements in walking capacity and physical functioning enhancing quality of life in people with PAD with IC and people without IC [36]. It is estimated that even small increases in physical activity for people with IC would significantly reduce all-cause mortality [12].

However, an international survey found only 30 % of vascular surgeons had access to supervised exercise programmes for patients with PAD, and of those who did have access, many referred less than half their patients [37]. Patient travel to facility-based supervised exercise sessions is burdensome, and drop-out rates are often high [38–40]. Lack of patient and physician awareness of the role of supervised exercise, and patient barriers of transportation and time contribute to this being an underused treatment [37, 41, 42]. For those surgeons who do not have access to refer to supervised exercise programmes, the usual option is to give brief advice to do walking [37].

However, people with IC have condition-specific barriers to walking that constrain participation. IC is an intense, cramping pain in the lower limbs, and some patients believe this pain is harmful [43], and have low confidence in their walking ability [44, 45]. Patients tend to be unclear about the causes and severity of PAD and the associated cardiovascular risk [43, 46, 47], and are unaware of walking recommendations and the mechanisms by which walking can improve PAD-specific and general health outcomes [43]. The surgical model of care can precipitate beliefs of an acute, treatable condition [43] and little personal control over health outcomes [44, 47]. Illness and treatment beliefs predict adherence to recommended levels of walking in people with PAD with very high accuracy [44]. A willingness to persist with activities given the concurrent pain, and a desire to be functionally competent, are also positively associated with physical mobility in people with PAD [45]. Adaptive self-management beliefs are therefore a key target to enable walking among people with PAD.

Behavioural counselling is a patient-centred approach that can promote adaptive illness- and exercise-related beliefs associated with sustained physical activity behaviour change in people with chronic conditions and cardiovascular risk [48, 49]. Preliminary evidence indicates that counselling can improve physical activity in people with PAD. A program combining weekly cognitive behavioral counselling and walking sessions demonstrated significant improvements in accelerometer assessed physical activity (mean difference 114.7 activity units, 95 % CI 12.82 to 216.5; *P* = .03); 6-min walk distance (mean difference 53.5 m, 95 % confidence interval [CI] 33.2 to 73.8; *P* < .001); Walking Impairment Questionnaire (WIQ) speed score (mean difference 10.4, 95 % CI 3.4 to 17.4; *P* = 0.004) and distance score (mean difference 11.1, 95 % CI 3.9 to 18.1; *P* = 0.003) [50]; and psychosocial indicators of satisfaction with physical functioning, pain acceptance and social functioning [51] relative to a health education control group. There were sustained improvements after 12 months in 6-min walk distance (mean difference 34.1 m, 95 % CI, 14.6 to 53.5; *P* < 0.001) and WIQ speed score (mean difference 8.8, 95 % CI, 1.6 to 16.1; *P* < 0.018) [52]. However, this approach was resource intensive, with weekly sessions delivered over 6 months. A more brief counselling intervention, involving two 1-h sessions of individualised behavioral counselling and two follow up telephone calls by a psychologist, demonstrated a significant increase in pain-free walking distance and daily walking in the intervention versus the control group (usual care plus non-walking related conversation during home visits) after four months, with a mean difference of 1575 pedometer steps/day (95 % CI, 732 to 2419) [53]. At 24 months, the intervention group completed on average 1630 steps/day (95 % CI, 495 to 2765) more than the control group, and significantly more participants in the control group had endovascular or open surgery than in the intervention group (OR 3.09 95 % CI 1.06 to 9.04; *p* = 0.037) [54]. However, as the counselling was delivered by a psychologist in patients’ homes, the wide scale applicability of this approach is not known.

## Methods/Design

### Aim and research questions

The primary aim of this study is to test the efficacy of brief behavioral counselling, delivered by allied health professionals, to increase physical activity in people with PAD. Secondary aims are to examine the effect of the counselling on functional capacity, revascularisation rate, clinical events, and resource use; and to perform an economic evaluation of the counselling intervention compared to usual care. This fills an important gap in the literature on PAD clinical management, and will address the following questions

- can brief behavioural counselling be delivered by health professionals who are not qualified psychologists, following training?
- can the brief behavioural counselling be effectively delivered in a variety of different centres by different professionals?
- is delivery of the brief behavioural counselling in a health care setting effective in achieving sustained physical activity behaviour change?

### Design and setting

This is a multicentre, prospective, randomised superiority trial with two parallel study groups: a control and an intervention group. The trial will be conducted in four cities across Australia. Study sites will include three specialist vascular management clinics (Townsville, Brisbane, Perth) and a University clinic (Sydney).

### Management

The trial will be overseen by a Trial Steering Committee comprising senior vascular investigators from the centers involved; and experts in trial management, physical activity behavior change, health economics and exercise physiology. This will be the main policy and decision-making committee. Trial coordination will be facilitated by the National Health and Medical Research Council (NHMRC) funded National Centre for Research Excellence in Peripheral Arterial Disease (NCRE-PAD). An independent data management center (CCRE therapeutics, Monash University) will provide a computer generated randomisation list. A study coordinator located at James Cook University will organise activities across the participating sites; assisting with local ethics reports and communication with the local sites (including uniformity of collection and reporting of data, central randomisation, data storage and processing). The training, competency assessment, and supervision of allied health professionals who will deliver the intervention and control group protocol; and the intervention fidelity assessment, will be undertaken by a clinical and health psychologist on the investigatory team (NB).

### Participants

Participants will be recruited from specialist vascular management clinics at The Townsville Hospital, The Royal Brisbane and Womens’ Hospital, and Fremantle Hospital Perth; and from referrals from vascular surgeons and general practitioners in Sydney. Participants will also be recruited from the investigators’ registry of participants involved in prior research studies who have agreed to be re-contacted.

People who express an interest in the study will be screened and enrolled by dedicated assessment staff either in person or by telephone. Inclusion criteria are

- Diagnosis of PAD by a vascular specialist, including patient-reported symptoms of IC and/or absence of lower limb pulses and/or resting ankle brachial pressure index (ABPI) <0.9 or >1.4 [23];
- Able to walk without assistance of another person
- No current or planned involvement in an organised exercise trial or program
- No currently planned peripheral vascular intervention
- Physician confirmed ability to participate in the trial (i.e. medically stable, able to walk and undertake physical activity, attend assessments and comprehend the behavioral counselling). Patients with severe PAD, such as rest pain, arterial ulceration or gangrene requiring urgent vascular intervention will be ineligible.

Exclusion criteria are

- Previous major lower limb amputation such as above or below knee
- Patients who report doing more than 120 min of moderate-vigorous structured exercise per week.

### Sample size calculation

We hypothesise that over a 4-month period the intervention will increase physical activity by ~50 %, evidenced by mean daily step counts of 5000 ± 3400 for those receiving the counselling intervention compared to 3300 ± 2200 for those randomised to the control group. We also hypothesise that these effects will be maintained at 12 and 24 months post intervention. The effect size and outcome results estimated are based on data from a previous trial [54]. We require 62 participants per group (power 90 %, alpha 0.05, within-subject between visit correlation 0.6) to detect the hypothesised increase in physical activity. Allowing for maintenance of this increase over 24 months, and a drop out of up to 40 %, we require 100 participants per group. We therefore will aim to recruit a total of 200 people.

### Randomisation

Eligible and consenting individuals who complete the baseline assessment will be randomly allocated to the control or intervention group. The allocation sequence will be computer-generated using a random number sequence and allocation will occur in block sizes up to 4 and will be stratified based on ABPI (<0.50, 0.50-0.69, 0.70-0.89, >1.4), study site, and gender. Allocation is concealed by using a central randomisation site, and different project staff for assessment and allocation/intervention delivery. Participants will be blinded to allocation and the investigators’ hypotheses. Intervention staff will conduct both intervention and control participant contacts, and therefore cannot be blinded to allocation. They will be instructed not to disclose the allocation status to either the participants or assessment staff.

### Study protocol and interventions

Recruitment and informed consent will be performed by project staff not directly involved in the patients’ care. Potential participants will be approached individually either by telephone, mail or in person; provided with the study information; and invited to participate. People who are screened as eligible and provide written consent will be randomised to either the intervention or control group.

**Fig. 1:**
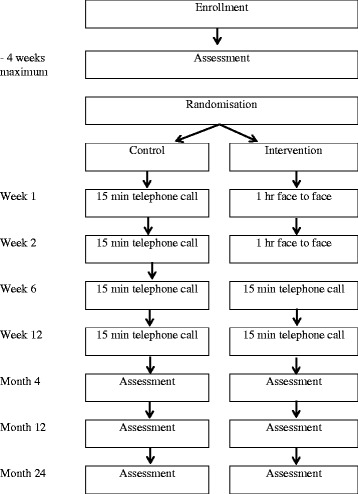
Study design, participant flow, and assessment schedule

An overview of the participant timeframe is presented in Fig. 1. Participants in both groups will receive ongoing medical care from their treating physicians, a written PAD management information sheet including advice to walk, and four individualised contacts from an allied health professional over 3 months (weeks 1, 2, 6 and 12).

The information sheet provides an overview of PAD (symptoms, contributing factors) with recommendations to stop smoking, eat a healthy diet, manage associated conditions such as diabetes and hypertension, and to walk for at least 40 min three times a week (see Additional file 1). The information includes instruction to walk until a moderate level of pain is experienced in the legs, rest until the pain subsides, and then resume [25].

The allied health professionals are required to be not a qualified psychologist e.g., nurse, dietician, exercise physiologist. They will be trained in the control and intervention protocol by the project psychologist in a face-to-face meeting (6 h) and given a written manual and summary participant contact protocol. They are required to achieve at least 80 % in a competency-based assessment administered two weeks before commencing participant contact. The assessment requires verbal descriptions of how they would use the study protocol in response to a series of vignettes reflecting participant case scenarios. During the study period, regular individual teleconferences (approximately monthly) will be held between the allied health staff and the psychologist, to discuss and problem-solve participant contact issues that arise. To assess intervention fidelity, all participant contacts will be audio recorded and a random selection of sessions (5 %) will be assessed at study conclusion by the psychologist against a protocol checklist.

#### Passive control group

Participants in the control group will receive four 15-min telephone calls, during which they will be asked open ended questions about PAD symptoms and general health and wellbeing (e.g., “How have you been since we last spoke?, How does your PAD impact on that?”, “How does that affect your PAD?”) and receive empathic non-specific responses. Project staff will respond to any specific queries on PAD management or physical activity by encouraging participants to read the written PAD management information sheet and talk with their treating physician. The combination of ongoing medical care from treating physicians and a written PAD management information is intended to represent usual care. The four telephone calls equalises the frequency of participant contacts between the control and intervention group. The telephone calls to control group participants may also mask allocation: participants in a no contact control group could determine allocation to a non-intervention condition.

#### Active intervention group

Participants in the intervention group will receive two 1-h face to face behavioral counselling sessions at a university or hospital site, and two 15-min telephone sessions. The counselling protocol used in this trial is based on the 5A Behavioral Counselling Framework, which is an evidence-based approach appropriate for physical activity change in people with chronic conditions and cardiovascular risk [55]. The key components of this approach as applied in this study are:

- Assessment: During the initial session, open ended questions and clarification probes will be used to understand patient perceptions of knowledge of PAD and benefits of walking for PAD, current walking (frequency and duration), and walking change (i.e., risks, barriers, enablers, outcome expectancies, feelings, reasons to change/not change). Subsequent sessions will focus on current walking (frequency and duration), and experiences with walking change (i.e., barriers, enablers, feelings, outcomes).
- Advice: Verbal advice will be provided to acknowledge pros and cons of walking, and confirm understanding and correct misperceptions about PAD and walking (e.g., PAD causes and consequences, interpretation of walking pain, benefits of walking vs. surgery for PAD). Clear and strong advice will be provided on the need to do regular walking to manage PAD symptoms and reduce the risk of serious health events.
- Agree: This will involve collaborative goal setting for walking i.e. graded goal setting (frequency and duration) towards recommendations to do three 40-min sessions per week of interval walking at maximum tolerable speed (i.e., moderate pain level with rest periods).
- Assistance: Discussion on action planning (when, where and with whom to do walking), motivational support (reflecting assessment information), self-regulation strategies, self-monitoring, social support, and identifying and problem solving barriers to walking (promoting coping, flexibility and control). This information will be individually tailored to participants’ interests, opportunities and abilities. The interventionist will provide praise, highlight effort towards achievement and gains, and promote adaptive interpretation of setbacks.
- Arrange: The next contact will be arranged where appropriate.

### Participant retention and withdrawal

Once enrolled, the study site will make every reasonable effort to follow the participant for the entire study period. Study site staff are responsible for implementing local standard operating procedures to optimise engagement and minimise attrition. To optimise engagement, contact and assessment times will be prearranged with participants on an individual basis. The allied health professionals will prearrange each contact i.e. the first contact will be organised at the assessment session, the second contact will be organised at the first contact etc. Assessment staff will arrange individualised assessment sessions by telephone.

The allied health professionals will be trained how to respond to participant ambivalence regarding subsequent contacts. The individualised pros and cons of engagement will be acknowledged and problems with engagement discussed. The importance of understanding the diverse spectrum of patient experiences with PAD, irrespective of patient perceptions of change, will be indicated. If participants request to discontinue contact, then participants will be asked if they are willing to engage with the assessment.

Participant non-attendance at face to face sessions or for telephone contacts will be followed up by the allied health professionals. Participant non-attendance at assessment sessions will be followed up by assessment staff. Non-attendance follow up will be primarily by telephone with a minimum of ten attempted contacts conducted at different times of day. Email or a single mail contact may also be attempted. If participants provide a secondary contact at the initial assessment, this will be used to determine if the participant is not available due to extenuating circumstances such as hospitalisation or an adverse event. Participant non-attendance for control/intervention contacts will be rescheduled as soon as possible after originally scheduled contact. If this is not possible within a 1–2 week period, then the contact will be identified as not occurring. The timing of following control/intervention contacts will be relative to the initial session. The assessment timing will remain constant relative to the first intervention/control contact. Participants who do not attend one assessment will be invited to attend subsequent assessment sessions unless they specifically request to withdraw from the study entirely.

Any physician prescribed or participant initiated concomitant care is permitted during the trial. Participants may request to withdraw from the study for any reason at any time. Should participants choose to withdraw, all efforts will be made to identify the reason for withdrawal.

### Participant safety

The behavior counselling intervention in this study is non invasive, low risk and should be well tolerated by the majority of participants. It is therefore unlikely that the trial would require stopping because of safety issues. Participant safety will be monitored through adverse event reporting via report forms. Adverse events may be identified by assessment or intervention staff during contacts, or spontaneously reported by participants. Definition of adverse events (AE) and serious adverse events (SAE) will be in line with the Good Clinical Practice Guidelines (GCPG) [56]. AEs will be defined as any unfavourable and unintended sign, symptom or disease that occurs in a participant whether it is considered to be study-related or not. A SAE will be defined as an untoward medical occurrence which results in death, requires inpatient hospitalisation or prolongation of existing hospitalisation, results in permanent or significant disability or is life threatening. All SAEs will be reported to the relevant Human Research Ethics Committee (HREC) as soon as possible following identification by study centers. The Trial Steering Committee will monitor SAEs and consider trial termination if events which appear related to the intervention are identified.

### Assessment

Assessments will be conducted at baseline and 4, 12 and 24 months by staff blinded to participant allocation. Each assessment time point will comprise two facility-based visits approximately one week apart, the first for 90 min, and the second for 60 min. At visit 1 (90 min) participants will provide a blood sample and complete physical activity and health-related quality of life questionnaires. The Short Physical Performance Battery (SPPB) and 6-min walk test (6MWT) will be undertaken. An assessment of ABPI will be performed. Smoking status, medical and surgical history, anthropometry, resting heart rate, brachial blood pressure and ambulatory ability will also be assessed. At the end of visit 1, participants will be fitted with an activPAL3™ accelerometer, which will be worn for seven consecutive days. At visit 2 (60 min), participants will return the activPAL3™ accelerometer, and complete additional physical activity and health related quality of life questionnaires. At baseline only, the six minute walk test is repeated at visit 2. Participants will be given the written PAD information sheet at the end of visit 2. Assessments at 4, 12 and 24 months will also include the recording of any study defined clinical events since the previous visit.

### Primary outcome measures

The primary outcome measure will be daily physical activity level or walking (i.e. steps per day). This will be assessed using an activPAL3™ accelerometer (PAL Technologies Ltd, Glasgow, United Kingdom) which is a triaxial accelerometer that assesses body position (i.e. sitting/lying and standing) and walking activity from accelerometer recordings at a sampling rate of 20 Hz and sensitivity of ± 2 g [57, 58]. The activPAL3™ unit will be wrapped in a nitrile sleeve, wrapped in waterproof dressing and attached to the right thigh of each participant using adhesive waterproof dressings (mid-line; 1/3 of the distance from the inguinal fold to the top of the patella). Recordings will commence at 06:00 on the following morning and continue for seven full days whereupon the activPAL3™ unit will be removed, returned to the investigators and the recording uploaded for analysis. Data are checked to ensure data has been recorded for the period, and if not, participants are asked to redo the assessment. The activPAL3™ has been reported to be reliable and valid in the assessment of walking, body posture, and sedentary behavior during free-living activity [57, 59, 60] and when used with older adults [61] and patients with IC [62].

### Secondary outcome measures

In addition to the primary measures of daily physical activity level and walking (steps/day), other outcome measures from the activPAL3™ will include daily sedentary (sitting/lying) time, number of sit-to-stand transitions, and energy expenditure (total number of kilocalories per day). Other secondary outcome measures using objective assessment will include:

- Ambulatory ability and lower body functioning: This will be assessed using the 6MWT, and the Short Physical Performance Battery (SPPB) which have previously been used as outcome measures in PAD intervention trials [36, 37]. The 6MWT assesses maximal cardiorespiratory fitness and correlates strongly with physical activity levels among people with PAD, and may therefore reflect walking capacity in everyday life [63]. The SPPB includes three objective tests of lower body function: A hierarchical test of standing balance, a timed 4-m walk and 5-timed repetitive chair stands. The SPPB strongly predicts loss of ability to walk 400 m [40], and is predictive of hospitalisation, nursing home admission and mortality [38, 39, 64]. It is a widely used and valid measure with established test-retest reliability [65].
- Event-based claudication index (EBCI) which is a measure of the ratio of walking bouts to upright time assessed from accelerometer data. Individuals with PAD have a greater EBCI than age matched controls, which indicates a fragmented stop/start walking pattern [47, 62].
- Vascular interventions including endovascular and surgical lower limb revascularisations and aneurysm repair.
- Clinical events including cardiovascular events, amputations, vascular and cardiac surgeries/interventions, musculoskeletal injury, other injuries or illness and death determined from patient reviews and hospital chart reviews, as described elsewhere [66, 67].
- Circulating pro-thrombotic markers, pro-inflammatory cytokines, lipids and RNA expression of circulating cells will be examined at the completion of the trial using stored blood samples obtained at each visit. EDTA and citrate plasma, serum and total blood collected into Paxgene tubes will be obtained at each assessment time. We have previously established methods to assess vascular biomarkers with good repeatability in our central laboratory [68–74].
- Measures of cardiovascular function including heart rate, blood pressure and ABPI assessed with standardised protocols as described elsewhere [75].
- Body composition including waist and hip circumference and body mass index assessed with standardised protocols as described previously [76].

Secondary measures using self-administered questionnaires include the following.

- Physical activity will be assessed using the International Physical Activity Questionnaire (IPAQ- short form) and the Physical Activity Scale for the Elderly (PASE). The IPAQ has four item groups that use a past 7 days recall period to ask about frequency (days/week) and time (mins/day) spent walking for at least 10 min at a time and in vigorous- and moderate-intensity activities; and time (mins/day) spent sitting on a week day. The instrument has good repeatability and demonstrated validity comparable with other self-report measures in diverse samples, settings and countries [77]. The PASE has 11 item groups that use a past seven days recall period to ask about frequency (never, 1–2, 3–4, 5–7 days) and average time/day (<1 h, >1- < 2 h, 2–4 h, >4 h) spent in sitting activities, walking, light sport or recreational activities, moderate sport or recreational activities, strenuous sport or recreational activities, and exercise to increase muscle strength and endurance; participation (yes/no) in domestic activities (light housework, heavy housework, home repairs, yard work, outdoor gardening and caring for another); time (hours/week) spent in volunteer work; and the degree of physical activity done as part of paid or volunteer work (mainly sitting, sitting or standing with some walking, walking with some light-moderate handling, walking and heavy manual work). Additional items will be used to also assess distance (number km) of walking and number of flights of stairs to enable Harvard Alumni Questionnaire scoring. The PASE has a good correlation with measures of lower body functioning and the 6MWT [78] and low PASE scores are predictive of cardiac-related mortality after coronary surgery [79].
- Sedentary behavior will be assessed using the SIT-Q-7d Questionnaire. This has 18 item groups that use a recall of the past seven days to assess average sitting time for meals (8 categories ranging from none to >1 h/day); for travel (to and from occupation, as part of occupation, and other purposes: 13 categories ranging from 0 to >7 h/day); as part of occupation (12 categories ranging from 0 to >8 h/day); for non-occupational screen-based activities (includes reclining for watching television, recreational computer use, playing computer games: 11 categories ranging from 0 to >8 h/day); and other activities (reading, household tasks, caring for others, hobbies, socialising, listening to music, other: 11 categories ranging from 0 to >7 h/day). Items also assess frequency (times/day) of interrupting sitting time during each of occupation and watching television; the time of going to sleep and getting up and duration of napping; and frequency of consuming each of 11 snack types while watching television (10 categories ranging from none to >5 times/day). The questionnaire has acceptable repeatability and good criterion validity for total sedentary time in otherwise healthy people [80].
- Walking ability will be assessed using the Walking Impairment Questionnaire (WIQ). This has 21 items that assess degree of pain and other difficulties (e.g., shortness of breath), difficulty in walking for a range of distances and speeds, and difficulty in climbing stairs. Responses are recorded on a 5-point Likert scale with options of none, slight, some, much, and very/unable. WIQ cut off scores appropriately classify walking performance (absolute claudication distance and peak walking time) assessed by a standardised graded treadmill test in people with PAD [81], and is a valid tool to detect improvement in daily walking ability in people with IC [82].
- Health-related quality of life (HRQoL) will be assessed with the disease-specific Intermittent Claudication Questionnaire (ICQ) [83] which has 16 items that use a recall of the past two weeks and a 5-point Likert scale for responses. Items ask about severity of leg pain (none to very severe); the extent to which leg pain limits a range of activities such as walking and crossing the road (not limited to totally limited); frequency of needing to stop walking (nil to >3x/day); and the extent to which the respondent has thought about, felt down about, worried about, had work interference from, had interference with hobbies and social activities from, and had interference doing errands from leg pain (all the time to none of the time). The questionnaire has acceptable test retest reliability, internal consistency and construct validity in people with intermittent claudication [83].
- Health-related quality of life will also be assessed using the PAD Quality of Life (PADQoL), the EQ-5D-5 L, and the SF36 (SF-36 v2) tools, which have previously been identified as useful for people with PAD [84–86]. The PADQoL has 38 items that ask about PAD-related issues and experiences such as activity restrictions; feelings of loss, vulnerability, burden, hope, and confidence; and impact on work, family and social activities. Responses are recorded on a 6-point Likert scale with options ranging from strongly agree to strongly disagree. The EQ-5D-5 L is a descriptive system of health-related quality of life states consisting of five dimensions (mobility, self-care, usual activities, pain/discomfort, anxiety/depression). The responses record five levels of severity (no problems/slight problems/moderate problems/severe problems/extreme problems) within a particular EQ-5D dimension. The SF-36 has 36 items and assesses eight health concepts [87]: limitations in physical activities because of health problems; limitations in social activities because of physical or emotional problems; limitations in usual role activities because of physical health problems; bodily pain; general mental health (psychological distress and well-being); limitations in usual role activities because of emotional problems; vitality (energy and fatigue); and general health perceptions. Two overall component scores can be derived to reflect physical and mental health. Items assess current functioning and during the past four weeks, and responses are provided on Likert type scales with three to five options.

To support an economic evaluation, cost information will also be collected. The following direct health care costs will be determined for every participant using data collected as part of the case report form (CRF):

- The resources associated with the interventions including the costs of training allied health professionals, the cost of delivering the behavior counselling intervention and the cost of monitoring changes in physical activity; These costs include both the salary cost of time spent by staff (pro rata, including on-costs) for the intervention training, implementation, and advisory sessions; administrative costs (e.g., telephone calls), location costs (rooms, utility costs), and travel costs of patients (e.g., time and financial outlay).
- The health services resources used for cardiovascular events will be assessed by data collected on any hospital admissions during follow-up including reason for admission, length of stay and type of vascular intervention performed. Overall, the cost of each participant’s continuous inpatient stay (CIS) will be estimated using the AR-DRG Grouper software licensed by Independent Hospital Pricing Authority (IHPA). This software takes into account key variables that impact on a patient’s CIS, such as principal and other diagnosis, length of stay, age and sex.
- For each medication prescribed during follow-up the associated costs will be estimated based on the unit costs of prescriptions determined from finance/purchasing department of the participating hospitals.

Some site-specific assessments will also be conducted (See Additional file 2).

### Data management and analyses

All study information will be stored securely at the study site. All participant information will be stored in locked cabinets in areas with limited access. All data will be identified by a coded identification number only to maintain participant confidentiality. Records that contain names or personal identifiers - such as case notes, consent forms or appointment records - will be stored separately from records identified by code number. Trial documents including study design, protocol, assessment manual, safety reporting forms and case report forms (CRF) and necessary software will be electronically shared with participating study centers.

Data recorded on printed CRFs will be scanned and emailed to the trial coordinating center along with the data file for the activPAL3™ accelerometer for assessment of data quality and central data entry. Data from the CRFs will be entered into a study database which incorporates data validation rules to reduce transcription errors and can only be accessed by authorised personnel. An extract of the database will be examined regularly and checked by the coordinating centre for data quality. Data entry is done by staff blinded to participant allocation.

Data from the activPAL3™ will be analysed using the manufacturer’s software (PAL Technologies Ltd, Glasgow, United Kingdom) to derive measures of physical activity and sedentary behavior. Data from the activPAL3™ will be uploaded and visually inspected for anomalies. Participant data will be considered valid if there is a minimum of 20 h per day and 5 days of wear time. Default settings of the activPAL3™ will be used to calculate 15-s epochs for each variable using: a minimum sitting and/or upright period of 10 s; a minimum walking cadence of 20 steps per minute; and energy expenditure values of 1.25 MET for sitting/lying, 1.4 MET for quiet standing, and 4 MET for walking cadences of >119 steps per minute.

Between group differences in physical activity (primary outcome) and other measures will be analysed using an intention-to-treat approach and repeated measures mixed models. All continuous data will be inspected for normality of distribution visually and statistically, and non-normally distributed data will be mathematically transformed if possible prior to use with parametric statistics. Covariates considered for use in these models include age, gender and any characteristics significantly different between groups at baseline which could potentially confound the variable of interest in each model. Predictors of changes in primary and secondary outcomes will be identified using simple and multiple regression models of relevant baseline characteristics (e.g., BMI and education). The incidence of revascularisation in the intervention and usual care control groups will be compared by chi-square test or logistic regression adjusted for relevant baseline characteristics if needed. Binomial logistic regression will be conducted to identify factors that predict revascularisation. Differences in time to revascularisation will be analysed by Kaplan Meier analysis and log rank test. Given the low risk of the behavior intervention planned in this study it is highly unlikely that the trial would require stopping and thus no interim analysis is planned. Relative effect sizes and 95 % Confidence Intervals for primary and secondary outcomes will be calculated. A *p* value of <0.05 will be accepted as statistically significant, given that all hypotheses to be tested have been formulated *a priori.*

The base case economic evaluation will adopt the perspective of the health sector, meaning that the data analysis will be restricted to including the costs and outcomes that directly impact upon the health sector. Two forms of analysis will be undertaken: a cost-effectiveness analysis and a cost-utility analysis. The cost-effectiveness analyses will compare the direct costs of the intervention (minus any cost savings) against the activity outcomes. An incremental cost effectiveness ratio (ICER) will be calculated as follows:

$$ \mathrm{ICER}=\left(\mathrm{C}1-\mathrm{C}0\right)/\left(\mathrm{E}1-\mathrm{E}0\right) $$

where C1 is the cost of the intervention (minus cost savings); C0 is the cost of the usual care; E1 is the effect from the intervention; and E0 is the effect of the usual care. C0 includes the costs associated with four separate patient appointments, which comprises of a general conversation, and on-going medical care. The difference between C1 and C0 is the incremental or additional cost being introduced to the health system. Similarly, the difference between E1 and E0 is the incremental effectiveness of the intervention. Within a sensitivity analysis, any working time lost will be converted into productivity costs, thus widening the perspective of the analysis to include some societal costs.

From the information above, three cost-effectiveness ICERs will be generated, as described below. For each ICER, the incremental cost of the intervention will be identical; whereas for effects, three activity outputs will be used. This analysis will then generate: (i) cost per additional 1000 steps/day walked; (ii) cost per additional 100 calories/day expended; and (iii) cost per additional minutes of moderate to vigorous intensity physical activity/day). This analysis will be performed at the 4-month post intervention and 12 and 24 month follow-ups.

The cost-utility analyses will be then be undertaken. The same direct costs will be included as before, however, in this case, the outcome measure will be changed to quality-adjusted life years (QALYs). Health utility scores will be generated for each participant by converting responses to HRQoL questionnaires into a single score using validated algorithms. The algorithm weights responses to HRQoL questions using population preferences and hence health utility score are also known as (preference-weighted) HRQoL. To estimate a quality-adjusted life year for cost-utility analysis, we will convert the EQ-5D and SF-36 into utility scores (the latter through the SF-6D) [88], and apply Australian utility weights for each [89, 90]. This permits cross-validation checks for the health utility values elicited and exploration of the sensitivity of the incremental outcome to choice of quality of life measure post-intervention. These analyses will be performed at the 12 month and 24 month follow-up. In Australia, a new intervention is generally considered to be cost-effective if the ICER, the mean cost per unitary change in mean utility is ≤ $50,000 [91, 92].

If the intervention is found to successfully improve health outcomes, and to result in reduced time lost from work, then the ‘perspective’ of the analysis would widen from the health sector to incorporate productivity savings. For those of working age, time lost from employment will be estimated using the World Health Organization’s Health and Performance Questionnaire (HPQ) [93]. Working time lost will be converted to productivity losses by using average Gross Domestic Product (GDP) per capita, adjusted pro rata. If individuals are made unemployed (rather than temporary sickness absence) then an assumption will be made regarding the length of time before that position is replaced by another person in the general population. A 90 fractional day period will be assumed, before replacement takes place. Any productivity losses would then be factored into the value of C1 above (reducing it), and the ICERs would be recalculated. This stage of the analysis would be reported separately as these potential productivity savings do not fall under the health care budget, and so is outside of the health sector funder’s perspective.

Uncertainty analysis will be conducted by bootstrapping patient level data and a value of information analysis (VOI) will estimate the need to undertake further research to inform whether the intervention is value for money [94]. All economic evaluation work will be implemented using STATA and Microsoft Excel® (Microsoft Corporation, Redmond, WA, USA).

Results will be disseminated via relevant scientific (e.g., journals, conferences), professional (e.g., newsletters), and public (e.g., media) forums. There are no plans to use professional writers.

## Discussion

People with PAD constitute a high-risk population with high rates of cardiovascular events and hospitalization; and greater associated costs compared to patients presenting with coronary artery disease alone [7]. Supervised exercise programs can improve maximal walking distance, pain symptoms and health-related quality of life [20, 28–31], but have limited accessibility [32]. Unsupervised home- or community-based exercise is a potentially attractive alternative to supervised exercise training, and can be effective in increasing walking capacity, community-based walking, and quality of life among people with PAD [28, 95, 96]. Components of successful interventions to promote unsupervised exercise in people with IC include self-monitoring, goal setting, and problem solving barriers [96]. Behavioral counselling typically includes these components, and there is preliminary evidence that brief behavioral counselling by a psychologist can promote sustained increases in walking in people with PAD [53, 54], but the generalisability of this to other health professionals is not known. In addition, little is known about the impact of such programs on cardiovascular disease risk [96].

This trial will test the efficacy and cost effectiveness of brief behavioral counselling delivered by allied health professionals in promoting physical activity and improving clinical outcomes in people with PAD. Using a range of allied health professionals to deliver the counselling across several settings will enhance wide scale applicability if it proves effective. This brief intervention could have a significant impact on PAD and how it is managed in clinical settings, and represent a pragmatic means to improve cardiovascular outcomes.

### Trial status

At the time of manuscript submission (October 2016) this study was ongoing. Recruitment and data collection commenced in Townsville in January 2015, and in Brisbane and Sydney in September 2015, and was pending in Perth.

### Trial sponsor

The Queensland Research Centre for Peripheral Vascular Disease; College of Medicine and Dentistry, James Cook University. Townsville QLD 4811, Australia.

### Protocol version

At the time of submission, the trial was using protocol version 5.3 (June 2016). Protocol amendments will be managed and disseminated by the Trial Coordinating Centre.

## Additional files

Additional file 1: PAD Information Sheet. (PDF 240 kb)
Additional file 2: Site Specific Additional Assessment. (PDF 177 kb)

## Abbreviations

6MWT: Six minute walk test
ABPI: Ankle brachial pressure index
AE: Adverse events
CI: Confidence interval
CRF: Case report form
EBCI: Event based claudication index
EDTA: Ethylenediamine tetraacetic acid
FBC: Full blood count
GCPG: Good clinical practice guidelines
GDP: Gross domestic product
HPQ: World Health Organisation Health and Performance Questionnaire
HREC: Human Research Ethics Committee
HRQoL: Health related quality of life
IC: Intermittent claudication
ICER: Incremental cost effectiveness ratio
ICQ: Intermittent Claudication Questionnaire
IPAQ: International Physical Activity Questionnaire
LOS: Length of stay
NCRE-PAD: (Australian) National Research Centre of Excellence in Peripheral Arterial Disease
NHMRC CCRE: (Australian) National Health & Medical Council Collaborative Centre for Research Excellence
NHMRC: (Australian) National Health & Medical Council
OR: Odds ratio
PAD: Peripheral arterial disease
PADQoL: Peripheral arterial disease quality of life questionnaire
PPAQ: Paffenbarger Physical Activity Questionnaire
QALY: Quality adjusted life year
RCT: Randomised controlled trial
SAE: Serious adverse events
SPPB: Short physical performance battery
WIQ: Walking Impairment Questionnaire
