# Supplementary material for: Efficacy of brief behavioral counselling by allied health professionals to promote physical activity in people with peripheral arterial disease (BIPP): study protocol for a multi-center randomized controlled trial
Source: BMC Public Health. 2016 Nov 9;16:1148. doi: 10.1186/s12889-016-3801-7 (PMC5103607; doi:10.1186/s12889-016-3801-7)
Supplement: Additional file 1: — PAD Information Sheet. (PDF 240 kb) [file 12889_2016_3801_MOESM1_ESM.pdf]

Peripheral Arterial Disease

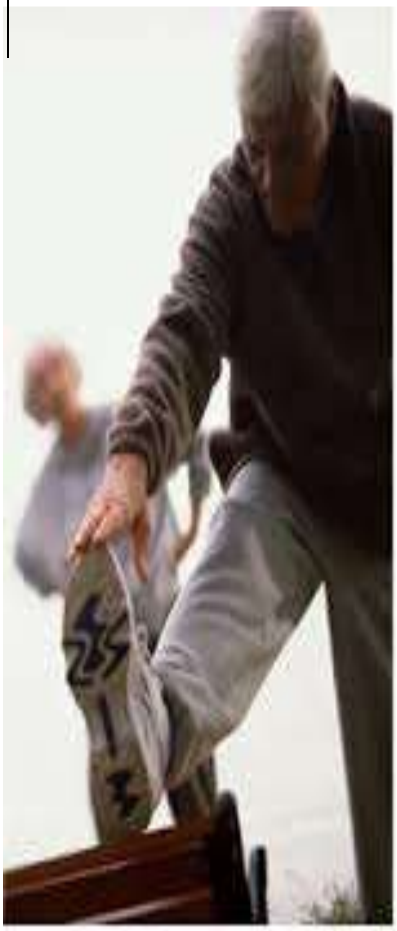

Peripheral Arterial Disease (PAD)

is a narrowing or blockage of the vessels that carry blood from the heart to the legs.

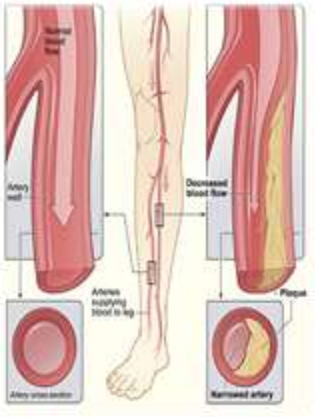

It is primarily caused by the build-up of plaque – a fatty deposit which limits blood flow.

PAD is a serious blood vessel disease, putting people at more than double the risk for a heart attack or stroke.

Symptoms

The typical symptom is pain which develops in the leg (calf, thigh, buttocks) with exertion such as walking or climbing stairs, and is relieved by resting. This pain varies and you may feel aching, cramping or tiredness. This pain is referred to as intermittent claudication.

Factors contributing to PAD:

- Smoking
- High blood pressure
- Diabetes
- High cholesterol
- Older age
- Lifestyle
  - → Inactivity
  - → Unhealthy diet
  - → High body weight
- Family history of heart disease or diabetes

What you can do

There is no cure for PAD. Therefore, the aim of management is to optimise blood flow and prevent the possibility of a serious health event in the future. This involves making lifestyle changes that can restore your mobility, decrease your risk for heart attack and stroke, and possibly save your life.

PAD symptoms can be managed by the following:

STOP SMOKING

Healthy eating

Manage associated conditions eg. diabetes, high blood pressure

WALKING

Walking is one of the best ways to manage PAD.
It is recommended that walking is done at least 3 times a week for 40 mins each time.
Ideally, you should walk every day.

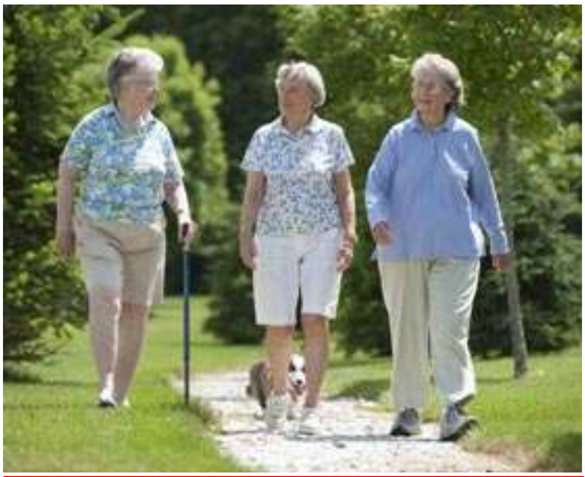

It may seem difficult, however walking is the **best way** to manage PAD.

Walk until the pain develops to a level of 3-4 on a scale of 5, then rest until the pain subsides. Carry on walking when the pain has eased.

The pain is not damaging to the muscles.

If you have any concerns or questions, please discuss these with your doctor
