# Supplementary material for: Efficacy of brief behavioral counselling by allied health professionals to promote physical activity in people with peripheral arterial disease (BIPP): study protocol for a multi-center randomized controlled trial
Source: BMC Public Health. 2016 Nov 9;16:1148. doi: 10.1186/s12889-016-3801-7 (PMC5103607; doi:10.1186/s12889-016-3801-7)
Supplement: Additional file 2: — Site Specific Additional Assessment. (PDF 177 kb) [file 12889_2016_3801_MOESM2_ESM.pdf]

Additional File 2: Site Specific Additional Measures

Brief behavioral counselling by allied health professionals to promote physical activity in people with peripheral arterial disease (BIPP)

At the Sydney site, the following additional assessments will be carried out at each assessment time point:

- Maximal exercise treadmill stress test with indirect calorimetry (Medgraphics PFX Ultima) will be used to assess peak aerobic capacity (VO_2peak). Participants will be monitored with a 12-lead electrocardiogram (ECG) (Quinton Q-Stress, USA) by a physician for safety. The treadmill (TM55, Quinton, USA) will be set to the participants habitual gait speed, with incline increasing by 2% every minute, up until a maximum of 24% grade, with the speed increasing by 0.5km/hr thereafter. Participants will be asked to walk until volitional fatigue, or until limited by symptoms, or if the supervising physician decides to terminate the test.
- Maximal strength and endurance muscle measurements will be obtained using the digital K400 Keiser pneumatic resistance machines (Keiser Sports health Equipment, Inc. Fresno, CA). Exercises performed include the seated leg press, bilateral knee extension, bilateral knee flexion, standing hip extension, calf raise, chest press, and seated row.
- Dual-energy X-ray Absorptiometry (DXA; Lunar Prodigy DXA Scanner and the enCORE software. GE Medical Systems Lunar, Madison, Wisconsin) will be obtained in a fasting condition to determine whole body and regional muscle, fat and bone mass. Regional scans of the lumbar spine as well as each hip will also be performed to determine bone mineral density at each site.

- Pulse Wave Velocity (PWV) Pulse Wave Analysis (PWA) Heart Rate Variability (HRV) will be determined using the SphygmoCor Unit and SphygmoCor software.
- 24-hour ambulatory blood pressure (TM-2430, A&D Co., LTD, Abingdon, Oxon, U.K.) will be assessed during waking hours (05:00 A.M to 10:00 P.M) and during sleeping hours (10:00 P.M to 05:00 A.M).
- Orthostatic hypotension (OH) will be determined with supine blood pressure and heart rate taken after the participant has been supine for at least 20 minutes, in a fasted state. Participants will then be asked to stand, with blood pressure and heart rate measurements taken 1- and 3minutes after standing. Blood pressure will be measured at the brachial level, with heart rate measured at the radial pulse for 15 seconds.
- Core self will be evaluated using the 12-item Core Self Evaluation Scale.[1] Responses are recorded on a 5-point Likert-type scale ranging from 1 (disagree strongly) to 5 (agree strongly). The scale measures a single factor that is the intersection of self-esteem, locus of control, generalized self-efficacy, and emotional stability.
- Stair climb power will be assessed. Participants will be asked to climb stairs as rapidly as possible to enable the calculation of Power (Watts). Power is calculated from the formula: P (watts) = (M × D) × 9.8/t Where: M = Body mass (kg), D = Vertical distance (m), t = Time (s) and, D = vertical height of the staircase = height of 1 step in meters × number of steps (if they are all the same height).
- Depression will be assessed using the Geriatric Depression Scale (GDS), which is a 30-item self-report assessment designed specifically to identify depression in the elderly. It has been validated against therapist ratings of depressive symptoms.[2]

At the Townsville site the following additional assessments will be conducted at each assessment time point:

- Pulse Wave Velocity (PWV) Pulse Wave Analysis (PWA) Heart Rate Variability (HRV) will be determined using the SphygmoCor Unit and SphygmoCor software.
- Skin autofluoresence will be measured using a non-invasive technique at the lower arm and the lower leg (AGE reader).
- Endothelial function will be measured at the finger using a non-invasive technique (EndoPAT).
- A dietary assessment will be administered as a structured interview and will consist of asking the patient to recall what food and drink has been consumed in the previous 24 hours. The information is then analysed using FoodWorks to find out the nutritional composition of the patient’s diet.

1. Judge T, Erez A, Bono J, Thoresen C. The Core Self-Evaluations Scale: development of a measure. Personnel Psychology. 2003;56(2):303–31.
2. Brink T, Yesavage J, Lum O, Heersema P, Adey M, Rose T. Screening tests for geriatric depression. Clin Gerontol. 2008;1(1):37–43.
